# Fissure sealant materials: Wear resistance of flowable composite resins

**DOI:** 10.15171/joddd.2016.031

**Published:** 2016-08-17

**Authors:** Sohrab Asefi, Solmaz Eskandarion, Shadi Hamidiaval

**Affiliations:** ^1^Orthodontist, Tehran, Iran; ^2^Assistant Professor, Department of Dental Materials, School of Dentistry, Shahid Beheshti University of Medical Sciences, Tehran, Iran; ^3^Assistant Professor, Department of Oral and Maxillofacial Radiology, Faculty of Dentistry, Lorestan University of Medical Sciences, Khorramabad, Iran

**Keywords:** Dental restoration wear, composite resins, pit and fissure sealants

## Abstract

***Background.*** Wear resistance of pit and fissure sealant materials can influence their retention. Wear characteristics of sealant materials may determine scheduling of check-up visits. The aim of this study was to compare wear resistance of two flowable composite resins with that of posterior composite resin materials.

***Methods.*** Thirty-five disk-shaped specimens were prepared in 5 groups, including two flowable composite resins (Estelite Flow Quick and Estelite Flow Quick High Flow), Filtek P90 and Filtek P60 and Tetric N-Ceram. The disk-shaped samples were prepared in 25-mm diameter by packing them into a two-piece aluminum mold and then light-cured. All the specimens were polished for 1minute using 600-grit sand paper. The samples were stored in distilled water at room temperature for 1 week and then worn by two-body abrasion test using "pin-on-disk" method (with distilled water under a 15-Nload at 0.05 m/s, for a distance of 100 meter with Steatite ceramic balls antagonists). A Profilometer was used for evaluating the surface wear. Data were analyzed with the one-way ANOVA.

***Results.*** Estelite Flow Quick exhibited 2708.9 ± 578.1 μm^2^ and Estelite Flow Quick High Flow exhibited 3206 ± 2445.1 μm^2^of wear but there were no significant differences between the groups. They demonstrated similar wear properties.

***Conclusion.*** Estelite flowable composite resins have wear resistance similar to nano- and micro-filled and micro-hybrid composite resins. Therefore, they can be recommended as pit and fissure sealant materials in the posterior region with appropriate mechanical characteristics.

## Introduction

The most common chronic disease during childhood is dental caries.^1^Occlusal cariescomprises80% of primary lesions of permanent teeth.^2^

Prevention of caries is important during development because caries may lead to speech, aesthetic and psychological problems, abnormal tongue habits and masticatory deficiencies.^3^

Monitoring of nutrition, regular oral hygiene, check-ups by a dentist and also use of fluoride products are preventive regimens for primary and permanent dental caries. Although fluoride can be effective in caries control for smooth surfaces, it is not very effective in preventing pit and fissures lesions. Therefore, pit and fissure sealant therapy is an effective method in preventing dental caries, especially during childhood and adolescent period. Although the occlusal surfaces of children’s teeth constitute almost 12.5% of total dental surfaces, 85% of total caries incidence is seen in these areas.^4^ Therefore, sealing of pits and fissures is an effective way for preventing occlusal dental caries.

During the past decades, a lot of materials have been suggested for this purpose like glass- ionomers, resin-modified glass-ionomers and resin-based composites such as compomer or flowable composite resins. The critical characteristics of these materials are good fluidity and low viscosity. Also, fissure sealant materials should have good bond strength and sealing ability to remain intact during the servicing period. Some of the disadvantages of sealants are microleakage, fracture toughness and wear.^5,6^

Wear resistance is an important necessity for sealant materials because it can help determine scheduling of check-up visits for evaluating sealant integrity and intactness.^7^

Low wear resistance for sealants may lead to restoration loss or fracture and also increased roughness and more plaque accumulation, which may result in caries development.^8^

Composite resin filler characteristics can influence composite resin wear. As the filler content of composite resin increases, we can expect more wear resistance in it in comparison to unfilled resins like pit and fissure sealant materials.^9^

In recent years, many studies have revealed that flowable composite resins can have bond strength and sealing ability similar to fissure sealant materials^10^ while they can have better mechanical properties.^11,12^ Also it was shown that wear resistance of flowable composite resins improved compared with universal composite resins in recent years.^13^

To determine the wear resistance of Estelite flowable composite resin as a fissure sealant material in the posterior region, we conducted an experimental study to compare the wear of these flowable composite resins with that of universal and packable ones. These flowable composite resins were utilized with mono-dispersing supra-nano spherical fillers and RAP technology (Radical Amplified Photopolymerization) for better resin polymerization. This can be useful in determining sealant servicing time and in planning checkup appointments.

## Methods

Ethics approval was not required because this was an in vitro study on dental materials. We conducted this in vitro study to compare wear resistance of two flowable composite resins: posterior universal and packable composite resins. A total of 35 specimens were prepared in 5 groups. The sample size was calculated based on the study of Yesil et al.^14^Minitab software program was used by considering α = 0.05, β = 0.2, a standard deviation of 0.06 and 0.1 as the significance level. Therefore, 7 specimens were included in each group. The commercial composite resins used in this study are presented in Table 1. The Table shows the type, size and volume of composite resin fillers in each group. Two-body abrasion test was the selected way for wear assessment and in this procedure we chose pin-on-disk method.^15^Disk-shaped specimens were prepared, measuring 25 mm in diameter; therefore, they were placed by a plastic carrier instrument or injected into customized two-piece aluminum molds with a gyrate space removed from the middle part. In order to pack and smooth the surface of composite resins, two thin glass blocks were used and the composite resins were curedfor 40 seconds from each side with a hand-held LED light-curing unit (Ultralume LED 5, Ultradent, UT, USA) in an overlapping manner for initial curing. The laboratory light-curing device, GC Labolight LV III (GC America Inc., Alsip, IL, USA), was used for 60 seconds on each side of the specimens for making sure of the same conversion effect. Surface roughness was standardized by polishing specimens with 600-grit sandpapers for 1 minute. This size of sandpapers has been used in some related articles;^14^ therefore, 600-grit size was used manually in all the groups. Soluble ingredients were removed by storing the specimens in distilled water for one week at room temperature. The specimens were worn by pin-on-disk device in Tribology Laboratory of Metallurgy School in Tehran University.

**Table 1:** Composites groups used in the study

**Material** **(Manufacturer)**	**Classification**	**Organic Matrix**	**Type of Filler**	**% Filler by Weight (Volume)**	**Mean Particle size of Filler**	**Shade**	**LOT**	**City/ Country**
**ESTELITE Flow Quick (Tokuyama dental Corp.)**	Nanofilled	Bis-GMA, UDMA, TEGDMA	Silica- Zirconia Supra-nano mono-dispersing spherical	71(53)	Microfiller: 0.4 Nanofiller: 0.07 µm	A3	019E11	Tokyo/ Japan
**ESTELITE Flow Quick High Flow (Tokuyama dental Corp.)**	Nanofilled	Bis-GMA, TEGDMA	Silica- Zirconia Supra-nano mono-dispersing spherical	68 (49)	Microfiller: 0.4 Nanofiller: 0.07 µm	OPA2	028EY0	Tokyo/ Japan
**Filtek P90 (3M ESPE)**	Microhybrid	Siloxane, Oxirane	Quartz	76	0.5 µm	C2	N212517	St. Paul/ USA
**Filtek P60 (3M ESPE)**	Microhybrid	Bis-GMA, UDMA, TEGDMA	Zirconium- silicate	71	0.6 µm	A3	N302776	St. Paul/ USA
**Tetric N-Ceram (Ivoclar Vivadent)**	Nanofilled	Bis-GMA, UDMA, TEGDMA	Glass microfiller	63.5	0.6 µm	A3	P75888	Schaan/ Liechtenstein

**Figure 1.**
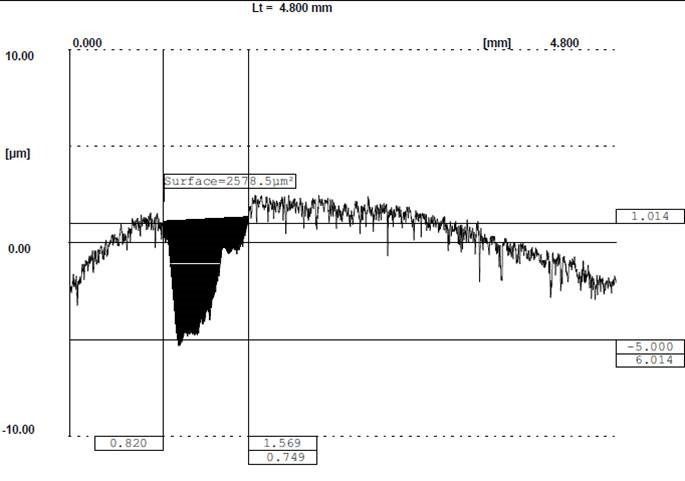
Surface roughness graph was drawn by profilometer. Cross section of worn surface area of each specimen can be calculated from this surface structure graph.

This tribometer is calibrated according to ASTM G99 standard periodically. In the pin-on-disk device, an antagonist material was installed on the mandrel of the device and the prepared disk-shaped specimens were fixed on the other compartment of the device. The disks were rotated by an electrical motor in a circular pattern and the antagonist was loaded under a predetermined force which was controlled by a digital load cell automatically. Wear procedure was stopped after the predetermined distance. The composite resin specimens were worn against Steatite ceramic balls as antagonists (Hoechst Ceram Tec, Wunsiedel, Germany).^16^ They measured 5 mm in diameter. The specimens were worn under a 15-N force and at 0.05 m/s for a 100-meter distance. In order to evaluate the wear of specimens, a Profilometer (T8000, Hommelwerke, Germany) was use. The Profilometer measured surface roughness in the direction of a radius of the disk randomly and indirectly constructed a surface structure graph in micrometer scale. We could able calculate the area of wear groove in each section by scanning one of the disk’s radii randomly. We found the wear area of each specimen as a groove in its cross-section based on surface graph which was constructed by the Profilometer (Figure 1). By multiplying this wear area by circle perimeter of the wear track, the wear of each specimen was calculated. Data was analyzed with one-way ANOVA and PASW18 software (PASW 18, SPSS, Chicago, IL, USA). Statistical significance was set at P <0.05.

## Results

Wear of each specimen was calculated by multiplying the wear area of the specimens from the Profilometer analysis by the perimeter of the experimental circle with a 22-mm diameter created by wear test device. Table 2 shows the mean of surface wear area for each group after two-body abrasion test. Wear of all the groups was similar, approximately 3585.35 μm.

**Table 2:** The means of surface wear area (μm^2^) of each group were measured by profilometry scanning. The groups were arranged by incremental wear tendency. There were no significant differences between the groups based on one-way ANOVA

**Material**	**Sample number**	**Mean**	**Std. Deviation**
**ESTELITE Flow Quick**	7	2708.9714	578.14781
**ESTELITE Flow Quick High Flow**	7	3206.0857	2445.16520
**Filtek P90**	7	3278.2142	1669.0809
**Filtek P60**	7	4335.9571	1879.8999
**Tetric N-Ceram**	7	4397.5714	3242.6515

P = 0.175

It was shown that the flowable composite resin (Tetric Flow) can have no microleakage and can be more efficient than resin sealant (Helioseal F) in sealing deep fissures.^18^ Also, flowable composite resins were more efficient and exhibited more penetration into shallow or wide fissures, although unfilled resins penetrates more into deep or narrow fissures.^19^ A meta-analysis in this field,^20^ and other clinical studies,^4,21,22^showed that flowable composite resins can have a retention rate similar to conventional pit and fissure sealants. Flowable composite resins were as effective as resin fissure sealants in caries prevention.^12^also In addition, it was shown that using flowable composites with total-etch technique resulted in better retention than conventional fissure sealants during a 24-months period and also better wear resistance and lesser surface porosity because of higher filler content.^12^ Therefore, it can be logical to use flowable composites as fissure sealant materials because of their more beneficial characteristics.

One-way ANOVA was used for inter-group comparisons. It did not show any significant differences between the experimental groups (P = 0.175). Estelite Flow Quick and High Flow composite resins exhibited wear patterns similar to those of posterior composite resins like P60 as packable and Tetric N-Ceram as universal nano-composite resins. Tukey multiple comparisons did not show any significant differences between each group pair (Table 3). Consequently, all the experimental groups did not show any significant differences in wear resistance, which can be very valuable for Estelite’s flowable composite resins compared with posterior packable composite resins.

**Table 3:** The P-value of Tukey multiple comparison tests between each paired group; none of the groups showed significant differences.

**Group**	**ESTELITE Flow Quick**	**ESTELITE Flow Quick High Flow**	**Filtek P90**	**Filtek P60**	**Tetric N-Ceram**
**ESTELITE Flow Quick**	—	1.000	0.999	0.802	0.771
**ESTELITE Flow Quick High Flow**	—	—	1.000	0.965	0.954
**Filtek P90**	—	—	—	0.976	0.967
**Filtek P60**	—	—	—	—	1.000
**Tetric N-Ceram**	—	—	—	—	—

## Discussion

Literature showed that permanent molars are susceptible to dental caries, especially during the 4-years period after eruption. Fissure sealant materials have not shown any significant differences in caries development or marginal discoloration from resin sealants but their major problems are higher wear and less retention; therefore, there has been a tendency to resin-based materials in order to increase wear resistance.^17^

Wear resistance is an important factor in clinical dental material selection because it can influence restoration service period. Composite resin wear can be influenced by several factors such as material characteristics, including filler content, silanization or degree of polymerization and other factors such as wear period, lubricating media, surface structure, temperature and contact stress.^23^

The more the composite resin is polymerized the more resistant it becomes against wear. In order to eliminate degree of conversion as a confounding factor in our study, we provided similar curing conditions for all the experimental groups.

Filler size, volume and hardness can influence wear characteristics of dental materials. By reducing the filler size, there is not a large organic matrix available between filler particles; consequently, it cannot be removed during wear procedure.^24^ Therefore, 0.1‒0.2 µm was considered as critical inter-filler space for dental composite resins.^25^

Our study results showed no significant differences between posterior packable composite resins and Estelite’s flowable composite resin. Sumino et al^13^ showed in a similar study that flowable composite resins with smaller filler size can have higher wear resistance than universal nanohybrid composite resins with larger prepolymerized fillers. They explained that prepolymerized filler particles, which are used to reduce polymerization shrinkage, cannot bond chemically to resin matrix with silanization; therefore, they can be exfoliated easily and resin matrix may be displaced rather than being really lost under stress. Finally, increased wear depth may be the final result of these composite resin types. They also reported that flowable composite resins have higher fracture toughness than packable composites and similar or higher than universal composite resins; therefore, this makes them capable of mounting greater resistance against crack propagation, which reduces wear susceptibility of flowable composite resins because of resistance to material break-down under mechanical stresses.

Another study^26^ compared wear resistance of one nanohybrid flowable composite resin (MI Flow, GC Corp.) with microfilled, micro-hybrid, hybrid, nanofilled and nano-hybrid composite resins in two- or three-body abrasion design. This flowable composite resin showed higher wear resistance in two-body abrasion test than other composite resins except for the microfilled one. This can be attributed to uniform and small filler particles (0.7 μm) in this flowable composite resin.

Estelite flowable composite resins utilize RAP technology and supra-nano mono-dispersing silica-zirconia fillers. The fillers are spherical and 0.4 and 0.07 µm in size with fine distribution. Their organic matrix in Flow Quick type (FQ) consists of BISGMA (2, 2-bis-[4-(methacryloxy-2-hydroxy-propoxy)-phenyl]-propane), TEGDMA (Triethylene glycol dimethacrylate) and UDMA (urethane dimethacrylate) but UDMA is not used in the type with high flow (HF).

The filler weight and volume percentage are 71% and 53%, respectively, in FQ composite resin; however, these measures are 68% and 49% in HF composite resin.

As shown in Table 1, Estelite flowable composite resins have high filler weight similar to posterior packable composite resins like Filtek P60 and also, their filler size is less than nanofilled composite resins like Tetric N-Ceram. Therefore, Estelite flowable composite resins improve mechanical properties by reducing filler size and increasing filler content, but they compensate increasing viscosity effect of smaller size and more filler content by using spherical filler shape to decrease friction between filler particles and interaction of matrix-filler. However, different filler types, spherical in Estelite composite resins, quartz and zirconium silicate in Filtek micro-hybrid composite resins and glass micro-filler in nanofilled Tetric N-Ceram composite resin, did not have a critical role in wear behavior of these composite types. Statistical analysis showed no significant difference between groups in wear resistance.

The first generation of flowable composite resins was created by reducing the filler content of hybrid composite resins in order to decrease material viscosity.^27^

There are three ways for increasing composite viscosity: increasing filler content, using irregular filler shapes, and incorporating glass fibers. Although no strong correlation has been shown between filler particle morphology and rheological properties, viscosity can be increased in the order of spheres, grains, plates and rods. Therefore, use of spherical filler shape, as they used in Estelite flowable composite resins, can increase filler content and also fracture resistance. In addition, round filler shape results in less friction because of interactions between filler particles or filler and matrix compared to irregular filler shapes used in most flowable composite resins. Adding nano-fillers to flowable composite resins increases composite resin viscosity because of increased filler surface area and also matrix- filler interaction or between filler particles; in addition, it improves the mechanical and flow characteristics.^28^

Sumino et al^13^ used wear resistance and flexural strength as two critical measurements for comparing flowable composite resins with universal composite resins as posterior restoration materials. Although wear resistance is one of the factors affecting the restoration durability, it will be better to consider other mechanical characteristics in future studies, which can influence the final decision on material selection. According to the results of this study and other similar studies,^18-22^, and among high flow ability and optimum mechanical properties like wear resistance, flowable composite resins can be suggested for use as a durable preventive restoration in permanent molars.

One of the limitations of this study was unavailability of three-body abrasion device in famous Iranian metallurgy schools, like Tehran, Elmosanat and Amirkabir Universities or their devices could not tolerate long period of wear tests which are proper for high wear cycles of dental restoration materials in the oral cavity. The high cost of wear test and the profilometer led to the withdrawal of SEM analysis. The profilometer analysis is reliable only for wear measurement; therefore, SEM analysis was not carried out because of financial limitations. It is suggested that proper three-body abrasive devices be designed for dental research. In addition, more financial support should be considered for research purposes so that researchers can carry out investigations with the use of more reliable and up-to-date devices with better financial support.

## Conclusion

It is logical to use some kind of flowable composite resins as pit and fissures sealant materials in preventive dentistry; these composite resins have better mechanical properties like higher wear resistance which lead to longer durability and reduce office and patient expenditure.

## Acknowledgments

We appreciate the Tribology Laboratory staff at Metallurgy School of Tehran University for their assistance in wear test and profilometer analysis.

## Authors’ contributions

SA contributed to the study concept, study design, literature review, experiments, data analysis and manuscript preparation. SE contributed to the study concept, literature review and manuscript preparation. SH contributed to the literature review, study experiments, data analysis and manuscript preparation. All the authors have read and approved the final manuscript.

## Funding

The study was self-funded.

## Competing interests

The authors declare no competing interests with regards to the authorship and/or publication of this article.

## Ethics approval

Not applicable.
